# Childhood Lead Exposure Linked to Apple Cinnamon Fruit Puree Pouches — North Carolina, June 2023–January 2024

**DOI:** 10.15585/mmwr.mm7328a2

**Published:** 2024-07-18

**Authors:** Melanie D. Napier, Alan Huneycutt, Carissa Moore, Chris Goforth, Marc Komlos, Veronica Bryant, Scott M. Shone, Larry D. Michael, Edward H. Norman

**Affiliations:** ^1^Division of Public Health, Children’s Environmental Health, North Carolina Department of Health and Human Services;^ 2^Division of Public Health, North Carolina State Laboratory of Public Health, North Carolina Department of Health and Human Services;^ 3^Division of Public Health, Environmental Health Section, North Carolina Department of Health and Human Services.

**Summary**

**What is already known about this topic?**

Lead exposure is toxic even at low levels, especially in young children. In North Carolina, investigations are performed to identify potential exposure sources for children with blood lead levels (BLLs) ≥5 *μ*g/dL.

**What is added by this report?**

During June–August 2023, routine testing identified four children in three unrelated North Carolina homes with BLLs ≥5 *μ*g/dL. Investigations identified WanaBana Apple Cinnamon Fruit Puree pouches as the likely exposure source. A collaborative multilevel response led to detection of approximately 500 cases of childhood lead exposure potentially linked to consumption of apple cinnamon purees nationwide. Voluntary recall of the implicated products prevented additional exposures.

**What are the implications for public health practice?**

Routine BLL testing of young children and environmental investigations can help identify emerging sources of lead exposure.

## Abstract

Lead exposure is toxic even at low levels, resulting in impairments that can affect a child’s lifelong success. In North Carolina, testing for lead is encouraged for all children at ages 1 and 2 years and required for children covered by Medicaid; investigations are performed to identify potential exposure sources for children with blood lead levels (BLLs) ≥5 *μ*g/dL. During June–August 2023, routine lead testing identified four asymptomatic North Carolina children with BLLs ≥5 *μ*g/dL. Home investigations identified only WanaBana brand apple cinnamon fruit puree pouches as a potential exposure source; product samples contained 1.9–3.0 ppm of lead. An expanded nationwide investigation led to identification of approximately 500 cases of childhood lead exposure believed to be linked to consumption of apple cinnamon purees, including 22 cases in North Carolina. Fewer than one half (45%) of the 22 North Carolina cases were among children covered by Medicaid. A coordinated multiagency communication strategy was implemented in North Carolina to notify consumers of the hazard and provide recommendations for preventing further exposure. The Food and Drug Administration issued a nationwide public health advisory on October 28, 2023; 2 days later, the manufacturer issued a voluntary recall. Routine testing of young children for lead exposure, combined with thorough environmental investigations, can identify emerging sources of lead exposure and limit further harm. 

## Introduction

North Carolina encourages testing of all children for lead at ages 1 and 2 years and requires testing for children enrolled in Medicaid. All blood lead test results for children aged <6 years are reportable to the North Carolina Department of Health and Human Services (NCDHHS) Childhood Lead Poisoning Prevention Program (CLPPP) (*1*). A child aged <6 years with two consecutive capillary or venous blood lead levels (BLLs) ≥5 *μ*g/dL within a 12-month period is considered to have a confirmed, reportable lead level and is eligible for a home investigation conducted by a registered environmental health specialist (field investigator) from the applicable county health department to identify the likely source of lead exposure.* When edible or consumer products are suspected as a source of lead exposure, environmental samples are collected from the home and analyzed by the North Carolina State Laboratory of Public Health (NCSLPH) Inorganic Chemistry Laboratory.^†^ Edible or consumer products with lead levels above North Carolina’s reportable limits (≥1.0 ppm for most spices and foods) are reported to the Food and Drug Administration (FDA). Medical providers of children with confirmed BLLs ≥5 *μ*g/dL are advised to use the North Carolina Clinical Follow-Up Schedule^§^ to monitor the child’s BLL and to provide additional case management as warranted. During June–August 2023, routine lead testing identified four asymptomatic children in three unrelated households with BLLs ≥5 *μ*g/dL who are the focus of this report, triggering home investigations to identify and remove sources of exposure. 

## Investigation and Results

### Household A

During June 2023, routine blood lead testing identified two siblings, aged 1 and 3 years, living in a western North Carolina county, each of whom had two consecutive BLLs ≥10 *μ*g/dL within a 12-month period (Figure). An environmental investigation conducted in July did not yield any potential sources as the likely cause of lead exposure. The field investigator suspected that a food item commonly consumed by both children could be the source, because children aged 1 year and 3 years have different hand-to-mouth behavior, yet the siblings’ BLLs rose simultaneously. On a food log, the parents recalled that both siblings ate WanaBana fruit puree pouches. In mid-August, the field investigator sent samples of apple cinnamon and apple banana flavor WanaBana fruit pouches taken from the home to the NCSLPH. Results obtained in September indicated that the apple cinnamon flavor contained 1.9 ppm lead^¶^; the North Carolina CLPPP was notified. On October 17, an initial report including laboratory results, packaging photos, lot numbers, and place of purchase (retailer A) was submitted to FDA.

**FIGURE:**
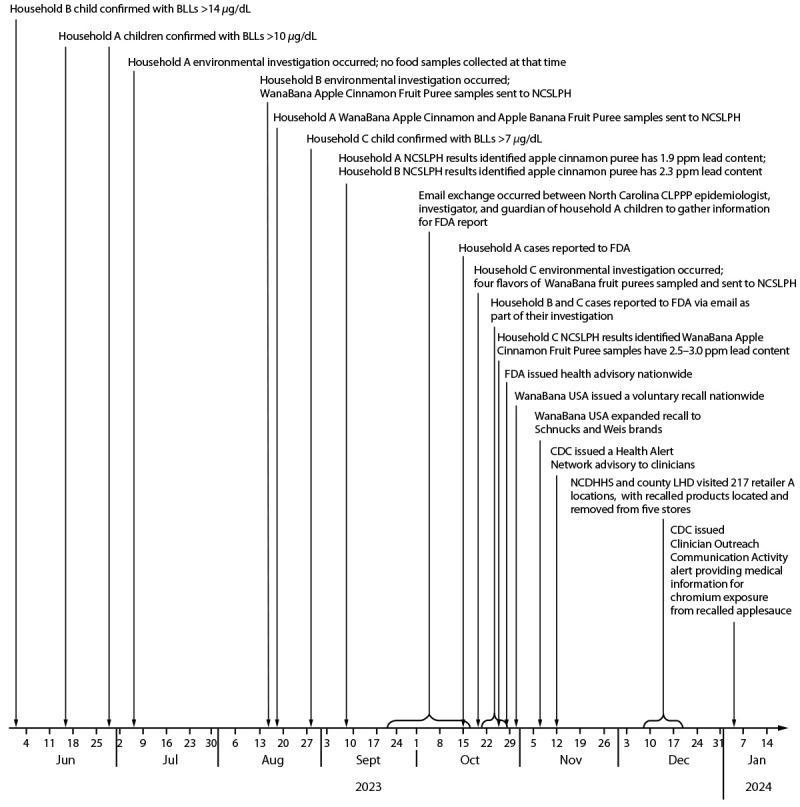
Response timeline* of an investigation of childhood lead exposure linked to consumption of WanaBana Apple Cinnamon Fruit Puree pouches — North Carolina, June 2023–January 2024 **Abbreviations:** BLLs = blood lead levels; CLPPP = Childhood Lead Poisoning Prevention Program; FDA = Food and Drug Administration; LHD = local health department; NCDHHS = North Carolina Department of Health and Human Services; NCSLPH = North Carolina State Laboratory of Public Health. * Confirmation of the case is based on the date of the second consecutive BLLs ≥5 *μ*g/dL. The order of the environmental investigations was based on when the site visit was conducted.

### Household B

During June 2023, a child aged 2 years living in a different western North Carolina county was identified through routine testing to have two consecutive BLLs >14 *μ*g/dL. An environmental investigation conducted in mid-August by the same field investigator who conducted the household A investigation did not identify any potential sources as the likely cause of lead exposure. However, when asked about food or spice consumption, the child’s parent mentioned that the child consumed applesauce pouches purchased from retailer A. A sample of WanaBana Apple Cinnamon Fruit Puree obtained from the home was sent to NCSLPH. In early September, NCSLPH reported that the sample contained 2.3 ppm lead.

### Household C

During August 2023, a child aged 1 year living in a third western North Carolina county was identified through routine testing to have two consecutive BLLs ≥7 *μ*g/dL. During preliminary interviews and water sample collection at the child’s home in September, none of the usual property-related lead sources were identified. During a home investigation in mid-October, the field investigator administered North Carolina CLPPP’s spice and home remedy survey.** The survey collects information that FDA requires to take public health action, including questions about spices, ceremonial powders, and alternative medicines, and is available in multiple languages. Using the survey, the investigator asked about consumption of cinnamon applesauce, which revealed that family members had purchased more than 90 pouches of four flavors of WanaBana fruit puree pouches for the child from three locations of retailer A in North Carolina and Kentucky. 

While at the home, the field investigator contacted the NCSLPH Inorganic Chemistry Laboratory to develop a comprehensive sampling plan. This plan included testing whole, unopened pouches of four flavors (apple cinnamon, pineapple and banana, apple and banana, and mango and banana) found in the home and the pouch material. Water, soil, and dust samples were also submitted. On October 24, NCSLPH reported that three different lot numbers of the apple cinnamon product contained lead in concentrations ranging from 2.5 to 3.0 ppm.

## Public Health Response

### Initial Response and Health Advisory

After FDA was alerted on October 17 that lead had been detected in a food product from household A, North Carolina public health officials and FDA worked together with county health departments to determine whether other children might have been exposed. North Carolina public health officials provided FDA with additional laboratory test results and the results of environmental investigations from households A, B, and C, including where affected lots were purchased, and collected product to test from retailer A locations across the state. North Carolina public health agencies also collaborated with the North Carolina Department of Agriculture and Consumer Services (NCDACS) laboratory, which provided retail product analysis, confirming NCSLPH results. Within 2 weeks, FDA confirmed North Carolina’s findings and issued a nationwide public health advisory. On October 28, North Carolina and FDA disseminated press releases urging consumers to dispose of contaminated products and contact their medical providers for testing (*2*,*3*). North Carolina public health officials notified all county health departments and the state’s child care licensing agency of the advisory. In accordance with North Carolina protocol for reporting food sample results during field investigations, households A, B, and C were advised to discard the apple puree products based on the initial NCSLPH product testing results. Follow-up testing indicated that BLLs among the affected children declined, adding confidence that the source had been identified.

### Voluntary Nationwide Product Recall

On October 30, WanaBana USA issued a voluntary nationwide recall of all lots of apple cinnamon fruit puree pouches that was expanded on November 9 to include private label brands Schnucks Apple Sauce with Cinnamon and Weis Cinnamon Apple Sauce (*4*). After the recall, NCSLPH continued to test WanaBana products collected statewide from homes and stores, demonstrating consistently elevated lead concentrations (1.9–5.8 ppm). On November 13, CDC issued a nationwide Health Alert Network advisory that indicated multiple states had reported to FDA potential cases of high BLLs among children consuming recalled cinnamon-containing applesauce and recommended that clinicians report cases to their local health authorities (*5*).

### Nationwide Investigation

After the recall, CDC launched a nationwide effort to systematically identify cases of BLLs greater than the CDC reference value of 3.5 *μ*g/dL among children associated with consumption of the implicated products^††^. By January 2024, a total of 22 cases among children in North Carolina (all with BLLs ≥5 *μ*g/dL, based on investigations going back to spring 2023) were identified and reported to CDC (Table). Of the 22 North Carolina cases, 10 (45%) were among children enrolled in Medicaid, and no typical sources of potential lead exposure were identified for any of the children with confirmed cases. On January 5, 2024, FDA reported that the source of lead in the involved products was cinnamon obtained from Ecuador, which also contained chromium in the form of lead chromate (*6*). A total of 519 cases nationwide were reported to CDC from state and local health departments as of March 22, 2024 (*6*). 

**TABLE:** Selected characteristics of children with confirmed blood lead levels* ≥5 *μ*g/dL and exposure to lead-contaminated WanaBana Apple Cinnamon Fruit Puree pouches (N = 22) — North Carolina, June 2023–January 2024

Characteristic	Investigated blood lead cases, according to CDC case definitions,^†^ no. (%)			
	Confirmed	Probable	Suspected	Total
**No. of cases (%)**	11 (50)	6 (27)	5 (23)	**22 (100)**
**Age, mos, mean (range)**	19 (12–37)	21 (12–26)	19 (12–33)	**20 (12–37)**
**Age, mos, median (IQR)**	15 (13–23)	22 (17–26)	18 (13–21)	**17 (13–25)**
**Male sex**	7 (64)	5 (83)	2 (40)	**14 (64)**
**Race**				
Asian	2 (18)	1 (17)	0 (—)	**3 (14)**
Black or African American	2 (18)	1 (17)	2 (40)	**5 (23)**
White	5 (45)	3 (50)	3 (60)	**11 (50)**
Unknown	2 (18)	1 (17)	0 (—)	**3 (14)**
**Hispanic or Latino ethnicity^§^**	0 (—)	1 (17)	1 (20)	**2 (9)**
**Enrolled in Medicaid**	3 (33)	3 (50)	4 (80)	**10 (45)**
**Initial BLL, *μ*g/dL, mean (range)**	15.2 (5.5–23.0)	9.1 (5.4–16.6)	7.1 (4.8–10.3)	**11.7 (4.8–23.0)**
**Confirmatory BLL, *μ*g/dL, mean (range)**	12.9 (8.1–23.5)	9.2 (5.2–15.9)	8.0 (5.5–12.8)	**10.8 (5.2–23.5)**
**Product lead level, ppm, mean (range)**	3.0 (1.9–5.8)	NA	NA	**3.0 (1.9–5.8)**

**Abbreviations: **BLL = blood lead level; NA = not available.

* In North Carolina, a child has a confirmed high BLL when they have a blood lead concentration of ≥5 *μ*g/dL determined by the lower of two consecutive blood tests within a 12-month period. The initial test might be from a capillary sample; however, the confirmatory test is preferably performed on a venous blood sample. Children with confirmed BLL 5–9 *μ*g/dL are eligible for a home investigation to determine the source of exposure. When BLLs are confirmed ≥10 *μ*g/dL, investigations are mandatory. Only those children eligible for an investigation (i.e., with BLLs ≥5 *μ*g/dL) are included in the data reported.

^†^ https://www.cdc.gov/lead-prevention/news/outbreak-applesauce-pouches.html?CDC_AAref_Val=https://www.cdc.gov/nceh/lead/news/lead-poisoning-outbreak-linked-to-cinnamon-applesauce-pouches.html

^§^ Persons of Hispanic or Latino (Hispanic) origin might be of any race but are categorized as Hispanic; all racial groups are non-Hispanic.

### North Carolina Recall Audit

The NCDHHS’s Environmental Health Section notified local food banks, child care center operators, and school food service managers of the recall through a statewide listserv. County health department staff members were advised to look for the recalled product during routine inspections of schools, child care centers, and institutional facilities. NCDHHS worked with NCDACS and the North Carolina Association of Local Health Directors’ leadership to ensure that recalled products were removed from retailer A stores. During 217 store visits conducted December 8–19 by county health department staff members, products were removed from five stores. 

## Discussion

Routine lead testing and environmental investigations in North Carolina resulted in the identification of a novel source of lead exposure that was ultimately linked to approximately 500 cases of childhood lead exposure nationwide, including 22 cases in North Carolina. In addition to following Centers for Medicare & Medicaid Services requirements for lead testing of Medicaid-enrolled children, CDC currently recommends focusing testing efforts on children having sociodemographic risk factors (e.g., being a racial or ethnic minority, living in a low-income household, or having environmental lead exposures) and those living in housing built before 1978.^§§^ However, fewer than one half of the North Carolina cases were among children enrolled in Medicaid, and no typical potential sources of lead exposure were identified as the likely cause for one half of the children, including those from households A, B, and C. This finding suggests that the recommendation for routine lead testing of all young children in North Carolina at ages 1 and 2 years might have led to detection of cases that would not otherwise have been identified and resulted in earlier identification and removal of a novel exposure source. 

Although lead-contaminated paint, water, dust, and soil are the most recognized lead hazards, other products have been found to contain lead, including candies, spices, ceremonial powders, and alternative medicines (*7*–*9*). As older houses containing lead-based paint are renovated or demolished, environmental sources have become less frequent. Awareness of other sources, such as spices adulterated with lead chromate, is important (*10*). 

This investigation highlights the potential benefits of broader routine blood lead testing for earlier detection of novel sources of lead exposure, such as foods and spices. Coordinated interagency collaboration and communication are essential for effectively detecting and responding to these events to prevent further harm.

## Footnotes

* An environmental investigation consists of visits to the home and potentially other addresses where the child regularly visits or spends time (defined by North Carolina General Statute Sect.130A–131.7[14]), such as child care facilities; the collection of water and environmental samples (dust, soil, and paint); and x-ray fluorescence analyzer readings in addition to an interview. The investigation usually concludes when the likely source is identified, which can take weeks of waiting for laboratory results or additional site visits. https://www.ncleg.net/EnactedLegislation/Statutes/PDF/ByChapter/Chapter_130A.pdf#page=108

† NCSLPH provides essential laboratory support to CLPPP partners. This support is accomplished through the American Industrial Hygiene Association’s Lead Assessment Program accredited testing of dust wipes, paint chips, and soil samples and through analytical screening of many other matrices, including spices, ceremonial powders, herbal remedies, cosmetics, toys, and foods. Food testing for lead is performed on inductively coupled plasma mass spectrometry instrumentation following Environmental Protection Agency method 6020B.

§
https://ehs.dph.ncdhhs.gov/hhccehb/cehu/lead/docs/ClinicalFollowUpSchedule_3.18.22.pdf

¶ FDA has developed draft guidance for action levels for lead in processed food intended for infants and young children: 10 ppb for fruits, vegetables (excluding single-ingredient root vegetables), mixtures, yogurts, custards and puddings, and single-ingredient meats; 20 ppb for root vegetables (single-ingredient); and 20 ppb for dry infant cereals. https://www.fda.gov/regulatory-information/search-fda-guidance-documents/draft-guidance-industry-action-levels-lead-food-intended-babies-and-young-children

**
https://ehs.dph.ncdhhs.gov/docs/forms/cehu/SpiceandHomeRemedySurveyFINAL-English-fillable.pdf

††
https://www.cdc.gov/lead-prevention/news/outbreak-applesauce-pouches.html?CDC_AAref_Val=https://www.cdc.gov/nceh/lead/news/lead-poisoning-outbreak-linked-to-cinnamon-applesauce-pouches.html

§§
https://www.cdc.gov/lead-prevention/php/news-features/updates-blood-lead-reference-value.html
